# A Retrospective Cohort Study on Diabetic Foot Disease: Ascertainment of Ulcer Locations by Age Group

**DOI:** 10.7759/cureus.28189

**Published:** 2022-08-19

**Authors:** Patrícia Rosinha, Miguel Saraiva, Lia Ferreira, Susana Garrido, André Carvalho, Cláudia Freitas, Cláudia Amaral, Luís Costa, Luís Loureiro, Rui Carvalho

**Affiliations:** 1 Endocrinology, Centro Hospitalar Baixo Vouga, Aveiro, PRT; 2 Endocrinology, Centro Hospitalar Universitário do Porto, Porto, PRT; 3 Orthopedics, Centro Hospitalar Universitário do Porto, Porto, PRT; 4 Vascular Surgery, Centro Hospitalar Universitário do Porto, Porto, PRT

**Keywords:** ageing, foot ulcer location, diabetes mellitus, elderly, diabetic foot ulcers

## Abstract

Background and aims

Diabetic foot ulcer location is a known independent predictor for cure with a better healing gradient proximal to distal. Although advanced age is one of the main factors associated with greater diabetic foot ulcer severity, there are no studies evaluating diabetic foot ulcer location specifically in the elderly population in an outpatient setting. This study evaluated diabetic foot ulcer location and age-group interactions in diabetic foot presentation.

Methods

A retrospective cohort study including adult patients with diabetic foot ulcers observed on their first visit to our center's Diabetic Foot Unit in 2018, divided into younger adults (YA) (18 to 64 years) and older adults (OA) (≥65 years).

Results

A total of 435 patients were included in the study with 159 (36.6%) in the YA, and 276 (63.4%) in the OA group.

Neuro-ischemic diabetic foot ulcers were more frequent in the OA group (71.4% vs 43.4%, p<0.001). The number of patients with a history of diabetic foot ulcers was lower in the OA group (18.1% vs 25.2%, p=0.03). A smaller proportion of forefoot diabetic foot ulcers (74.9% vs 86.2%, p=0.007) and plantar location diabetic foot ulcers (9.4% vs 24.5%, p<0.001) occurred in the OA group.

By univariate logistic regression analysis, we found two associations with older age: proximal (odds ratio (OR) 2.09 (1.23-3.53), p=0.006), and non-plantar (OR 3.13 (1.82-5.37), p<0.001) diabetic foot ulcer location. After adjusting for potential confounders in a multivariate analysis, older age lost the association to more proximal (OR 1.72 (0.94-3.15), p=0.081) and non-plantar (OR 1.78 (0.83-3.77), p=0.133) diabetic foot ulcer location.

Conclusions

There are essential age differences in diabetic foot ulcer presentation. The OA group more frequently presents neuro-ischemic diabetic foot ulcers with more proximal and non-plantar locations.

## Introduction

Diabetic foot disease is a chronic complication of diabetes mellitus (DM) defined as foot ulceration associated with neuropathy and different grades of ischemia and infection, whose incidence has been increasing in proportion with the worldwide prevalence of DM [1,2]. Diabetic foot ulcers are important not only because of the high mortality associated with them but also due to the reduction in quality of life and inherent costs [2,3].

The improvement of living conditions and advances in science and medicine in recent years have led to an increase in average life expectancy, with subsequent aging of the population [4]. It is known that during the physiological process of senescence, important changes occur, namely loss of muscle mass and function, increased joint stiffness, reduced range of motion, and changes in gait/balance, phenomena that can be translated into mobility limitation and considerable functional disability [5].

Previous studies also have shown that advanced age is one of the main factors associated with greater severity of diabetic foot ulcers [1,6]. There is also evidence to demonstrate that diabetic foot ulcer location is a known independent predictor for cure with a better healing gradient proximal to distal [7]. No studies evaluating diabetic foot ulcer location specifically in the elderly population in an outpatient setting have been published.

Thus, our study's aim was to evaluate diabetic foot ulcer location and age interactions in diabetic foot presentation in order to better understand the diabetic foot ulcer location effect on prognosis over different age groups.

The results of this study were previously presented as a meeting abstract at the International Diabetes Federation Congress 2021 on December 6 to 11, 2021.

## Materials and methods

This is a retrospective observational study including 583 adult patients with diabetic foot ulcers observed on their first visit to the Diabetic Foot Unit of Centro Hospitalar Universitário do Porto, a tertiary care unit in Northern Portugal, during the year 2018. A total of 148 patients were excluded: 59 with no active diabetic foot ulcer and 89 lacking information on clinical records. The final sample of 435 patients was divided into two groups by age at diabetic foot ulcer presentation i.e., younger adults (YA) at 18 to 64 years, and older adults (OA) ≥ 65 years.

Data concerning demographic/clinical information, aspects related to diabetic foot ulcers, and their classification were collected from the electronic clinical record and entered into a database without any patient identifiers. Smoking habits were considered if there was a history of current or previous use. The value of glycated hemoglobin (HbA1c) was determined at the first visit using an HbA1c analyzer. Diabetic foot ulcer location was assessed anatomically (categorized as forefoot and midfoot or above) and according to the surface (plantar and non-plantar) and lateralization (right and left). For diabetic foot ulcer classification, each ulcer was graded/scored using three different scales: the perfusion, extent, depth, infection, sensation (PEDIS)/Infectious Diseases Society of America (IDSA) classification; University of Texas system classification; and Wagner’s classification (Table 1) [8,9].

**Table 1:** Diabetic foot ulcer classification scales DFU: Diabetic foot ulcer, IDSA: Infectious Diseases Society of America, PEDIS: Perfusion, extent, depth, infection, sensation

DFU classification scales	Grade/Stage	Description
PEDIS/IDSA	Grade 1	Ulcers without signs of infection (purulence or erythema, pain, tenderness, warmth or induration)
	Grade 2	Mild infection: the presence of at least two signs of infection (cellulitis <2 cm around the ulcer, infection limited to skin/subcutaneous tissue, and no other complications)
	Grade 3	Moderate infection (cellulitis >2 cm, streaking, deep tissue abscess, gangrene, involvement of muscle/tendon/joint/bone)
	Grade 4	Presence of systemic signs of infection or metabolic instability (fever, chills, tachycardia, hypotension, confusion, vomiting, severe hyperglycemia, acidosis, or azotemia)
University of Texas	Stage A	No infection or ischemia
	Stage B	Infection present
	Stage C	Ischemia present
	Stage D	Infection and ischemia present
	Grade 0	Fully epithelialized pre- or post-ulcerative lesions
	Grade 1	Superficial wound
	Grade 2	Wound penetrates to tendon or capsule
	Grade 3	Wound penetrates to bone or joint
Wagner	Grade 0	Closed lesion with deformation or cellulitis
	Grade 1	Superficial ulcer of the skin or subcutaneous tissue
	Grade 2	Ulcer that extends into tendon, bone, or capsule
	Grade 3	Deep ulcer with osteomyelitis or abscess
	Grade 4	Gangrene of toes or forefoot
	Grade 5	Midfoot hindfoot gangrene

The study protocol was in conformance with the World Medical Association’s Helsinki Declaration and was approved by the Ethics Committee of Centro Hospitalar Universitário do Porto (approval number: 2021.160 (131-DEFI/134-CE)). Informed consent was waived by the Ethics Committee based on the retrospective nature of the study and full data anonymization. 

Data analysis was performed using the Statistical Package for Social Sciences (SPSS) version 20 (IBM Corp., Armonk, NY, USA). Categorical variables are presented as frequencies and percentages and continuous variables as means and standard deviations (SD) or medians and interquartile ranges (IQR) for variables with skewed distributions. Normal distribution was checked using the Shapiro-Wilk test or skewness and kurtosis, as appropriate. Categorical variables were compared with Pearson’s chi-square test or Fisher’s exact test. Continuous variables were compared with the t-test for independent samples or the Mann-Whitney U test (if skewed distribution). Binary logistic regression was used to evaluate the presence of an association between older age and diabetic foot ulcer location, by adjusting for possible confounders. Anatomic location of diabetic foot ulcers and surface of diabetic foot ulcers were considered dependent variables in separate analyses. Independent variables included in diabetic foot ulcer surface analysis were the study group (YA/OA) and the following potential confounders: gender, level of education, dyslipidemia, nephropathy, and cerebrovascular disease. Independent variables included in diabetic foot ulcer anatomic location analysis were the study group (YA/OA) and the following potential confounders: gender, smoking habits, and motor autonomy. All reported p-values are two-tailed, with p<0.05 indicating statistical significance.

## Results

A total of 435 patients were included in this study with 159 (36.6%) in the YA (18-64 years) and 276 (63.4%) in the OA (≥ 65 years) group. The percentage of elderly people aged 75 or over was 61.6% in this sample.

The results are presented in the following Tables. Table 2 shows that the younger group had statistically more men (78.6% vs 52.5%, p<0.001), more smokers (49.1% vs 24.6%, p<0.001), more mobility (84.3% vs 60.1%, p<0.001) and higher levels of education (42.8%/33.3%/6.3% vs 72.5%/9.8%/1.1%, p<0.001). Type 2 DM was more common in older individuals (93.8% vs 78.6%, p<0.001), with a higher mean DM duration (21.4 ± 11.9 vs 16.1 ± 9.4 years, p<0.001) and a lower median HbA1c value (7.4% (IQR 2.1) vs 8.2% (IQR 2.8), p=0.001). The OA group had a statistically higher percentage of hypertension (81.2% vs 69.8%), dyslipidaemia (73.2% vs 59.1%), nephropathy (30.8% vs 20.8%), cerebrovascular disease (31.5% vs 13.8%), and ischemic heart disease (23.6% vs 13.8%). The prevalence of retinopathy was similar in both groups (39.1% vs 45.3%, p=0.467). There were no significant differences in the percentage of patients on insulin (51.8% vs 56.6%, p=0.541), statin (55.1% vs 49.7%, p=0.145), or anti-aggregation (43.5% vs 36.5%, p=0.116) treatment (Table 2).

**Table 2:** Demographic description of the sample by age group HbA1c: Glycated hemoglobin, IQR: Interquartile range, OA: Older adults, SD: Standard deviation, YA: Younger adults *Pearson's chi-square, ^a^ - independent samples t-test, ^b^ - Fisher's exact test, ^c^ - Mann-Whitney U test

	Total (n=435)		YA (n=159)		OA (n=276)		p-value
Gender, n (%)							
Male	270	(62.1)	125	(78.6)	145	(52.5)	<0.001*
Female	165	(37.9)	34	(21.4)	131	(47.5)	
Smoking habits, n (%)	146	(33.6)	78	(49.1)	68	(24.6)	<0.001*
Motor autonomy, n (%)	300	(69.0)	134	(84.3)	166	(60.1)	<0.001*
Level of education, n (%)							
≤4 years	268	(61.6)	68	(42.8)	200	(72.5)	<0.001^b^
5-12 years	75	(17.3)	53	(33.3)	22	(9.8)	
>12 years	13	(3.0)	10	(6.3)	3	(1.1)	
Dyslipidaemia, n (%)	296	(68.0)	94	(59.1)	202	(73.2)	0.001^b^
Under statin treatment, n (%)	231	(53.1)	79	(49.7)	152	(55.1)	0.145*
Hypertension, n (%)	335	(77.0)	111	(69.8)	224	(81.2)	0.011^b^
Type of diabetes mellitus, n (%)							
Type 1	41	(9.4)	31	(19.5)	10	(3.6)	<0.001*
Type 2	384	(88.3)	125	(78.6)	259	(93.8)	
Other	3	(0.6	1	(0.6)	2	(0.8)	
Under insulin treatment, n (%)	233	(53.6)	90	(56.6)	143	(51.8)	0.541*
Diabetes mellitus duration (years), mean ± SD	19.46 ± 11.35		16.1 ± 9.4		21.4 ± 11.9		<0.001^a^
HbA1c (%), median (IQR)	7.6 (2.3)		8.2 (2.8)		7.4 (2.1)		0.001^c^
Retinopathy, n (%)	180	(41.4)	72	(45.3)	108	(39.1)	0.467*
Nephropathy, n (%)	118	(27.1)	33	(20.8)	85	(30.8)	0.036^b^
Cerebrovascular disease, n (%)	109	(25.0)	22	(13.8)	87	(31.5)	<0.001*
Ischemic heart disease, n (%)	87	(0.2)	22	(13.8)	65	(23.6)	0.012*
Under antiplatelet therapy, n (%)	178	(40.9)	58	(36.5)	120	(43.5)	0.116^b^

The YA group more often had a past history of diabetic foot ulcers (25.2% vs 18.1%, p=0.03) (Table 3). The number or type of previous amputation did not differ significantly between groups (80.0%/18.5%/1.5% vs 86.1%/12.6%/1.3%, p=0.301; 14.4%/2.5%/1.9%/0% vs 6.5%/2.2%/2.5%/1.4%, p=0.055). Neuropathic diabetic foot ulcers were more common in the YA group (56.6% vs 28.6%, p<0.001). A greater proportion of both forefoot and plantar location diabetic foot ulcers (86.2% vs 74.9%, p=0.007; and 24.5% vs 9.4%, p<0.001, respectively) occurred in the YA group. No differences were found in terms of diabetic foot ulcer evolution time, lateralization, or previous course of antibiotic therapy (Table 3).

**Table 3:** Description of aspects related to diabetic foot ulcers by age group DFU: Diabetic foot ulcer, IQR: Interquartile range, TF: Transfemoral, TM: Transmetatarsal, TTP: Transtibioperoneal, OA: Older adults, YA: Younger adults *Pearson's chi-square, ^b ^- Fisher's exact test, ^c^ - Mann-Whitney U test

	Total (n=435)		YA (n=159)		OA (n=276)		p-value
Past history of DFU, n (%)	90	(20.7)	40	(25.2)	50	(18.1)	0.030*
Number of previous amputations, n (%)							
0	306	(70.3)	108	(80.0)	198	(86.1)	0.301^b^
1	54	(12.4)	25	(18.5)	29	(12.6)	
2	5	(1.1)	2	(1.5)	3	(1.3)	
Type of previous amputation, n (%)							
Minor (fingers)	41	(9.4)	23	(14.4)	18	(6.5)	0.055^b^
Minor (TM)	10	(2.3)	4	(2.5)	6	(2.2)	
Major (TTP)	10	(2.3)	3	(1.9)	7	(2.5)	
Major (TF)	4	(0.9)	0		4	(1.4)	
Type of DFU, n (%)							
Neuropathic	169	(38.9)	90	(56.6)	79	(28.6)	<0.001*
Neuro-ischemic	266	(61.1)	69	(43.4)	197	(71.4)	
Precipitating factor, n (%)							
Shoes	111	(25.5)	45	(28.3)	66	(23.9)	0.009*
Trauma	72	(16.6)	34	(21.4)	38	(13.8)	
Burn	11	(2.5)	6	(3.8)	5	(1.8)	
Pressure	8	(1.8)	0		8	(2.9)	
Other	35	(8.0)	15	(9.4)	20	(7.2)	
Unknown	198	(45.5)	59	(37.1)	139	(50.4)	
Evolution time of DFU (weeks), median (IQR)	4.0 (6.0)		4.0 (6.0)		4.0 (6.0)		0.297^c^
Anatomic location of DFU, n (%)							
Forefoot	343	(78.9)	137	(86.2)	206	(74.9)	0.007*
Midfoot or above	91	(20.9)	22	(13.8)	69	(25.1)	
Surface of DFU, n (%)							
Plantar	65	(14.9)	39	(24.5)	26	(9.4)	<0.001*
Non-plantar	370	(85.0)	120	(75.5)	250	(90.6)	
Lateralization of DFU, n (%)							
Right	219	(50.3)	76	(48.7)	143	(52.6)	0.482*
Left	209	(48.0)	80	(51.3)	129	(47.4)	
Previous course of antibiotic therapy, n (%)	219	(50.3)	83	(52.2)	136	(49.3)	0.615*

Table 4 summarizes diabetic foot ulcer classification by age group according to PEDIS infection, University of Texas, and Wagner classifications. There were no differences between groups in the percentage of patients in grade 1 (66.9% vs 70.7%) and grade ≥ 2 (33.1% vs 29.3%) in the PEDIS scale, nor in the stages and grades in the University of Texas classification. However, 68% of patients presented with a grade 1 ulcer as per the PEDIS scale and had a stage A and a grade 1 ulcer as per the University of Texas classification (60% and 79.1%, respectively) (Table 4).

**Table 4:** Diabetic foot ulcer classification by age group OA: Older adults; PEDIS: Perfusion, extent, depth, infection, sensation; YA: Younger adults *Pearson's chi-square

	Total (n=435)		YA (n=159)		OA (n=276)		p-value
PEDIS infection grade, n (%)							
Grade 1	296	(68.0)	105	(66.9)	191	(70.7)	0.446*
≥ Grade 2	131	(30.1)	52	(33.1)	79	(29.3)	
Stage of University of Texas classification, n (%)							
A	261	(60.0)	96	(61.1)	165	(61.1)	0.226*
B	100	(23.0)	43	(27.4)	57	(21.1)	
C	32	(7.4)	8	(5.1)	24	(8.9)	
D	34	(7.8)	10	(6.4)	24	(8.9)	
Grade of University of Texas classification, n (%)							
0	20	(4.6)	5	(3.2)	15	(5.6)	0.625*
1	344	(79.1)	127	(80.9)	217	(80.4)	
2	28	(6.4)	10	(6.4)	18	(6.7)	
3	35	(8.0)	15	(9.6)	20	(7.4)	
Wagner classification, n (%)							
Grade 0	17	(3.9)	3	(1.9)	14	(5.2)	0.559*
Grade 1	338	(77.7)	127	(80.9)	211	(78.4)	
Grade 2	26	(6.0)	9	(5.7)	17	(6.3)	
Grade 3	32	(7.4)	13	(8.3)	19	(7.1)	
Grade 4	13	(3.0)	5	(3.2)	8	(3.0)	

In the univariate logistic regression analysis, we found two associations with older age: proximal diabetic foot ulcer location (OR 2.09 (1.23-3.53), p=0.006) (Table 5), and non-plantar diabetic foot ulcer surface (OR 3.13 (1.82-5.37), p<0.001) (Table 6). After adjusting for potential confounders in a multivariate analysis, older age lost the association both to more proximal (OR 1.72 (0.94-3.15), p=0.081), and non-plantar (OR 1.78 (0.83-3.77), p=0.133) diabetic foot ulcer location.

**Table 5:** Univariate and multivariate logistic regression analysis of diabetic foot ulcer location (forefoot/midfoot or above) after adjustment for possible confounders CI: Confidence interval, DFU: Diabetic foot ulcer, DM: Diabetes mellitus, HbA1c: Glycated hemoglobin, OR: Odds ratio

	OR crude	CI 95%	p-value	OR adjusted	CI 95%	p-value
Older group (equal/above 65 years)			0.006			0.081
Yes	2.086	1.232-3.531		1.717	0.936-3.150	
No	1			1		
Gender			0.005			0.469
Male	1			1		
Female	1.949	1.221-3.111		1.246	0.687-2.262	
Smoking habits			0.039			0.376
Yes	1			1		
No	1.746	1.029-2.961		1.341	0.700-2.570	
Motor autonomy			<0.001			0.003
Yes	1			1		
No	2.936	1.724-5.000		2.334	1.330-4.096	
Level of education			0.281			
≤4 years	1.411	0.755-2.637				
>4 years	1					
Dyslipidaemia			0.946			
Yes	1					
No	0.981	0.568-1.695				
Hypertension			0.405			
Yes	1					
No	1.287	0.710-2.332				
Type of DM			0.620			
Type 1 or other	0.816	0.365-1.823				
Type 2	1					
DM duration (years)	1.005	0.984-1.025	0.661			
HbA1c (%)	0.892	0.753-1.056	0.185			
Retinopathy			0.071			
Yes	1					
No	0.640	0.394-1.040				
Nephropathy			0.652			
Yes	1					
No	0.652	0.526-1.495				
Cerebrovascular disease			0.629			
Yes	1					
No	0.878	0.519-1.486				
Ischemic heart disease			0.087			
Yes	1					
No	0.621	0.359-1.072				
Past history of DFU			0.463			
Yes	1					
No	1.257	0.682-2.319				
Previous amputation			0.767			
Yes	1					
No	0.903	0.459-1.775				
Previous course of antibiotic therapy			0.062			
Yes	1					
No	1.567	0.978-2.512				

**Table 6:** Univariate and multivariate logistic regression analysis of diabetic foot ulcer surface (plantar/non-plantar) after adjustment for possible confounders CI: Confidence interval, DFU: Diabetic foot ulcer, DM: Diabetes mellitus, HbA1c: Glycated hemoglobin, OR: Odds ratio

	OR crude	CI 95%	p-value	OR adjusted	CI 95%	p-value
Older group (equal/above 65 years)			<0.001			0.133
Yes	0.320	0.186 - 0.550		0.562	0.265 - 1.191	
No	1			1		
Gender			0.002			0.147
Male	1			1		
Female	0.359	0.189 - 0.681		0.557	0.252 - 1.230	
Smoking habits			0.324			
Yes	1					
No	1.350	0.744 - 2.449				
Motor autonomy			0.099			
Yes	1					
No	2.018	0.876 - 4.650				
Level of education			0.035			0.433
≤4 years	0.510	0.273 - 0.953		0.739	0.346 - 1.575	
>4 years	1			1		
Dyslipidaemia			0.001			0.144
Yes	1			1		
No	2.527	1.428 - 4.471		1.676	0.838 - 3.353	
Hypertension			0.742			
Yes	1					
No	0.884	0.426 - 1.837				
Type of DM			0.483			
Type 1 or other	0.706	0.267 - 1.866				
Type 2	1					
DM duration (years)	0.990	0.966 - 1.015	0.427			
HbA1c (%)	0.933	0.775 - 1.123	0.466			
Retinopathy			0.453			
Yes	1					
No	1.242	0.706 - 2.185				
Nephropathy			0.045			0.198
Yes	1			1		
No	2.043	1.017 - 4.103		1.789	0.738 - 4.337	
Cerebrovascular disease			0.016			0.167
Yes	1			1		
No	2.595	1.190 - 5.657		2.201	0.720 - 6.733	
Ischemic heart disease			0.408			
Yes	1					
No	1.359	0.657 - 2.811				
Past history of DFU			0.070			
Yes	1					
No	0.560	0.298 - 1.050				
Previous amputation			0.294			
Yes	1					
No	0.675	0.324 - 1.405				
Previous course of antibiotic therapy			0.358			
Yes	1					
No	1.282	0.755 - 2.177				

## Discussion

In this study, there was a higher prevalence of diabetic foot ulcers (63.4% vs 36.6%) in the elderly. We also found a lower proportion of males and a higher prevalence of comorbidities (namely dyslipidemia and hypertension) and type 2 DM in advanced age. In terms of ulcer-related factor results, there was a higher prevalence of neuro-ischemic diabetic foot ulcers and a smaller proportion of forefoot and plantar location diabetic foot ulcers in the older group. In the univariate logistic regression analysis, we found associations between older age and both proximal and non-plantar diabetic foot ulcer location even though these associations did not persist after adjusting for potential confounding factors.

The higher prevalence of neuro-ischemic diabetic foot ulcers in the older group is in line with the expected atherosclerosis continuous progress during aging [10,11]. In terms of location, proximal and non-plantar diabetic foot ulcers occurred more frequently in the older group, both with predictably lower healing rates according to the literature [7]. These aspects combined with their greater vulnerability, lower average life expectancy, and considerable age group heterogeneity reinforce the need to individualize the approach and treatment of diabetic foot ulcers in this age group [4]. 

The only study that we know of evaluating ulcer location and age group was published by Dörr et al. in 2021. However, patients in this study were hospitalized while our patients were seen in the outpatient setting and had less advanced disease [12]. When comparing our results with those of Dörr et al., they may, at first glance, seem contradictory as it was suggested that ulcers tend to move from the plantar and hindfoot to the forefoot and toes with increasing age [12]. Both results can be reconciled if we consider the substantial differences between the samples: one corresponded to hospitalized patients with diabetic foot ulcer infection and ours evaluated predominantly patients with uninfected diabetic foot ulcers observed in an outpatient setting. Thus, the key to explaining the disparity in outcomes appears to be the presence and severity of the infection. Although the most frequent diabetic foot ulcer location in the elderly is more proximal and non-plantar, infection is more frequently diagnosed in plantar and more distal ulcers. In our point of view, the most plausible explanations for this could be that the plantar and distal regions of the foot are more prone to trauma and therefore enable an easier "gateway" for infectious agents [13].

Furthermore, logistic regression analysis results suggest the presence of a potential confounding factor underlying the loss of association with more proximal diabetic foot ulcer location in the multivariate analysis. Thus, we admit that most of the confounding effects may have resulted from the patient's lack of autonomy, a factor that maintained its association with the more proximal location of diabetic foot ulcers in the multivariate analysis and that may have increased the risk of developing ulcers, especially proximal pressure-linked ones, or from other factors not considered in the analysis (for example vascular supply). We cannot assure that a part of the ulcers evaluated could not correspond to pressure-linked ulcers in individuals with DM, whose pathophysiology and risk factors differ. This topic has been debated. Our results support an overlap between diabetic foot ulcers and some pressure-linked ulcers in the elderly. Moreover, we consider that older age might have lost its association with both more proximal and non-plantar diabetic foot ulcers in the multivariate analysis due to the limited sample size.

This study is the first, to our knowledge, to assess diabetic foot ulcer location according to age group in an outpatient and predominantly non-infected setting. Moreover, we provide a complete assessment of diabetic foot ulcer severity based on the three most used and widely accepted diabetic foot ulcer classification systems [9]. However, a few limitations should be noted. Firstly, its retrospective nature accounts for the existence of missing values in some of the studied variables. We also acknowledge that there are inter-observer differences in the use of diabetic foot ulcer classification systems, which, despite evidence of a moderate inter-observer agreement between scales [9], might have led to a classification bias. Finally, it is also worth mentioning that vascular studies underlying the classification of diabetic foot ulcers were not included.

## Conclusions

In conclusion, elderly individuals are a very special population in several aspects, namely in diabetic foot ulcer presentation and location. Our results showed that this group is more prone to non-plantar and proximal diabetic foot ulcer location on univariate analysis, both with predictably lower healing rates according to literature, even though these associations did not persist in the multivariate analysis. We hope that these results may represent an advance for better prevention and management of diabetic foot ulcers in older individuals with diabetes.
